# Bioreactor-Based Online Recovery of Human Progenitor Cells with Uncompromised Regenerative Potential: A Bone Tissue Engineering Perspective

**DOI:** 10.1371/journal.pone.0136875

**Published:** 2015-08-27

**Authors:** Maarten Sonnaert, Frank P. Luyten, Jan Schrooten, Ioannis Papantoniou

**Affiliations:** 1 Prometheus, Division of Skeletal Tissue Engineering, KU Leuven, Leuven, Belgium; 2 Department of Materials Engineering, KU Leuven, Heverlee, Belgium; 3 Department of Development and Regeneration, Skeletal Biology and Engineering Research Center, KU Leuven, Leuven, Belgium; Universidade do Porto, PORTUGAL

## Abstract

The use of a 3D perfusion culture environment for stem cell expansion has been shown to be beneficial for maintenance of the original cell functionality but due to several system inherent characteristics such as the presence of extracellular matrix, the continued development and implementation of 3D perfusion bioreactor technologies is hampered. Therefore, this study developed a methodology for harvesting a progenitor cell population from a 3D open porous culture surface after expansion in a perfusion bioreactor and performed a functional characterization of the expanded cells. An initial screening showed collagenase to be the most interesting reagent to release the cells from the 3D culture surface as it resulted in high yields without compromising cell viability. Subsequently a Design of Experiment approach was used to obtain optimized 3D harvest conditions by assessing the interplay of flow rate, collagenase concentration and incubation time on the harvest efficiency, viability and single cell fraction. Cells that were recovered with the optimized harvest protocol, by perfusing a 880 U/ml collagenase solution for 7 hours at a flow rate of 4 ml/min, were thereafter functionally analyzed for their characteristics as expanded progenitor cell population. As both the *in vitro* tri-lineage differentiation capacity and the *in vivo* bone forming potential were maintained after 3D perfusion bioreactor expansion we concluded that the developed seeding, culture and harvest processes did not significantly compromise the viability and potency of the cells and can contribute to the future development of integrated bioprocesses for stem cell expansion.

## Introduction

As the field of tissue engineering evolves towards clinical applications, the development of well characterized bioprocesses to provide consistent production of tissue engineered (TE) advanced therapy medicinal products (ATMPs) becomes imperative. However, at current, the production of such ATMPs consists of a series of discrete manual unit operations ranging from progenitor cell isolation from donor biopsies, to cell expansion and differentiation to achieve those numbers needed for therapy and functional TE construct development. Although preliminary studies using these manual methodologies have demonstrated the potential of TE ATMPs for *in vivo* tissue regeneration [1, 2], closed and integrated bioprocesses should be developed to reduce the dependence on operator expertise and minimizing risk of contamination. The use of bioreactors is considered to be essential for the successful clinical introduction of novel ATMPs in these aspects [3, 4]. Next to contributing to the development of automated, controlled and monitored processes, bioreactors also enable the use of 3D cell culture substrates which were hypothesized to have beneficial effects on the characteristics of the expanding cell population such as enhanced maintenance of the original cell phenotype [5–9].

The use of perfusion bioreactors, incorporating 3D open porous inert and rigid scaffolds as 3D culture substrate for cell expansion, has been associated with significant advantages concerning the identity and potency of the resulting cell population [10]. In previous studies the ability of cells to grow into the third dimension leading to 3D culture surface with filled pores, has been demonstrated [11]. During 3D growth, cells secrete extracellular matrix (ECM) depending, amongst others, on the flow rate employed for cell culture [11, 12]. Even though the presence of a supportive ECM has been shown to possess significant advantages concerning maintenance of the potency of the expanded cells [13–16], cell recovery is significantly impaired, requiring dedicated process development and optimization.

Detachment or dissociation of cells from the culture surface with subsequent retention of cell quality is therefore equally important as cell attachment and proliferation, given that the product of interest in cell therapy applications is the cell itself [17, 18]. Despite reports of adverse effects on cell characteristics [19–21], trypsin is one of the most widely used reagents for cell recovery and was already used for the recovery of cells from microcarrier based expansion systems [22–24] as well as for the digest of primary tissues, although often in combination with other enzymes which specifically target the collagen containing fraction of the ECM [10, 25]. Additionally, various optimization studies for collagenase-based digestion of primary tissues such as cartilage are available indicating the feasibility of a trypsin free approach although no detailed reports are available regarding the recovery of cells from 3D culture surfaces [26–28].

Functional characterization of the expanded and recovered cell population is imperative to assess the relevance of the developed processes. Current approaches focus mainly on the *in vitro* characterization which enables the potential classification of the expanded population as being an adult mesenchymal stromal stem cell population [10, 11, 22, 24, 29–31]. However, the final goal of these expansion processes is to obtain a progenitor cell population which can contribute to the development of an *in vivo* functional tissue. Therefore not only the post expansion characterization but also the functional *in vivo* assessment of the expanded cell population is critical [31].

In this work we expanded human periosteum derived cells (hPDCs) in a 3D flow-through perfusion bioreactor [11] and monitored their growth non-destructively using the Presto Blue metabolic assay [32]. Upon confluence, as was confirmed by contrast enhanced X-ray nano-computed tomography (CE-nanoCT), a range of cell recovery reagents was assessed. Subsequently the most efficient harvest reagent was selected and for the harvest protocol was further optimized for the reagent concentration, flow rate used for detachment and harvest time leading to maximal cell viability and harvested single cell yield based on a Design of Experiment (DoE) approach. Finally, the functionality of the expanded progenitor cell population was defined using a combination of *in vitro* and *in vivo* proliferation, differentiation and bone forming assays.

## Materials and Methods

### Human periosteum-derived cells (hPDCs)

hPDCs were isolated from periosteal biopsies obtained from 4 different donors (age 11, 13, 14 and 17, equal distribution of gender) as described previously and pooled for further use [33]. This procedure was approved by the ethics committee for Human Medical Research KU Leuven (ML7861). Patient informed written consent was provided by the legal guardian. hPDCs were expanded in Dulbecco’s modified Eagle’s medium with high-glucose (Life Technologies) containing 10% fetal bovine serum (FBS, Gibco), 1% sodium pyruvate (Life Technologies) and 1% antibiotic–antimycotic (100 units/mL penicillin, 100 mg/mL streptomycin, and 0.25 mg/mL amphotericin B; Life Technologies), further mentioned as culture medium (CM). The cells were seeded at 5,700 cells/cm^2^ and passaged at 80%–90% confluency. Cell expansion was performed in standard cell culture conditions (relative humidity: 95%, 5% CO_2_, 37°C).

### Ti6Al4V scaffolds

For 3D perfusion bioreactor cell expansion, additive manufactured Ti6Al4V scaffolds (Ø = 6 mm, h = 6 mm) [34, 35] were used as described before [11, 12, 32, 36]. The total volume of the scaffolds was 166 ± 3 mm^3^, the available volume 130 ± 5 mm^3^ and the available surface 7.5 ± 0.6 mm^2^ as determined with μCT [37]. Scaffolds were cleaned and prepared for experiments as described before [11, 12, 32, 36].

### Scaffold seeding and culture

Prior to the 3D culture experiments cells were harvested at passage 6 using Triple Express (Life Technologies) and drop-seeded onto the scaffolds at a final density of 120,000 cells per scaffold [11, 12, 32, 36]. Scaffold-cell constructs were incubated overnight in standard culture conditions resulting in an initial cell density of 17,100 cells/cm^2^ or 700,000 cells/cm^3^.

For cell expansion, seeded constructs were inserted in an in-house developed perfusion bioreactor system consisting of an interconnected bioreactor chamber and a medium reservoir [11, 12, 32, 36]. A total volume of 10 ml CM was perfused at a constant flow rate of 1 ml/min. CM was refreshed every two days by attaching a new medium reservoir containing 10 ml fresh CM.

### Presto Blue measurement

To monitor cell growth, a Presto Blue (PB, Life Technologies) measurement was performed every second day on quadruplicate samples as described earlier for a period of 17 days [32]. 5 ml of a 9% solution was perfused for 2 hours at 1 ml/min after which three 100 μl samples were taken from each unit and the fluorescent signal was measured with a Synergy HT Multi-Mode Microplate Reader (Biotek) using an excitation wavelength of 544 nm and an emission wavelength of 590 nm [32]. The measured fluorescent signal was expressed as arbitrary fluorescent units (FU).

### Calcein Acetoxymethyl staining

A Calcein acetoxymethyl (AM) staining was used to qualitatively visualize the distribution of live cells by optical fluorescent microscopy. At day 3, 8 and 15, representative cell loaded scaffolds (based on PB measurements) were imaged. Constructs were rinsed with 1 ml phosphate buffered saline (PBS) after which they were incubated in the staining solution (0.5 μl of a 4 mM Calcein AM in anhydrous dimethylsulfoxide solution, Life technologies) for 20 min in normal cell culture conditions. The constructs were imaged using a Leica M165 FC microscope.

### Contrast enhanced nano computed X-ray tomography of cell expansion constructs

Cell—ECM structure developed during the expansion (further mentioned as neo-tissue) was visualized using contrast enhanced CE-nanoCT [11, 12, 38]. Prior to staining constructs were fixed in a 4% paraformaldehyde solution (Sigma) for 2 hours. To enable visualization of the neo-tissue, constructs were stained with a 60% Hexabrix 320 solution (Guerbet) for 20 min [11, 12]. A Phoenix NanoTom S (GE Measurement and Control Solutions) with a 180 kV/15 W high-performance nanofocus X-ray tube was used with a tungsten target, which was operated at a voltage of 90 kV and a current of 170 μA. An aluminum and copper filter, both 1 mm thick, were used to reduce beam hardening and metal artefacts. The exposure time was 500 ms, a frame averaging of 1 and image skip of 0 were applied, resulting in a scanning time of 20 min. The obtained radiographic images were reconstructed using Phoenix Datos|X (GE Measurement and Control Solutions). The reconstructed images had an isotropic voxel size of 3.75 μm [11, 12].

### Cell harvest

For the use of the StemPro Accutase Cell Dissociation Reagent (Life Technologies) or 0.05% Trypsin–ethylenediaminetetraacetic acid (EDTA) solution (Life Technologies) the bioreactor circuits were first rinsed with Phosphate buffered saline (PBS, Life Technologies) to remove all FBS remnants. Cell harvest using the Accutase solution was performed at a flow rate of 1ml/min up to one hour at 37°C or up to 6 hours at room temperature (incubation times and temperatures according to the manufacturer’s instructions for cell recovery and tissue dissociation). The trypsin solution was applied up to 40 min at 37°C at 1ml/min. For the collagenase IV solution (Life Technologies) a 440U/ml solution in CM was perfused at 1ml/min up to 13 hours at 37°C as was also performed for the initial digest of the periosteum [39]. Harvest efficiency was determined based on DNA measurements on the scaffolds after harvest and on the resulting cell suspension as described further. Scaffolds that did not receive any harvest treatment were used as a control. All conditions were performed in triplicate on constructs expanded for 13 days.

A DoE approach was used to further optimize the cell harvest procedure using the Collagenase IV solution. In order to determine the influence of incubation time, concentration and flow rate on the cell yield and the viability of the harvested cell population a 3-level, 3-parameter fractional factorial design was used resulting in 9 combinations of the aforementioned parameters as shown in Table 1. The assessed range of flow rates was selected based upon pervious work where we showed that no significant influence of the fluid flow could be observed on cell proliferation between 0.04ml/min and 4ml/min [11]. As the preliminary screening experiment for Collagenase IV showed no significant increase in cell yield after 7 hours this was selected as the maximal incubation time. Additionally, no negative influence of the used setup was observed on cell viability. Therefore, the assessed preliminary concentration was selected as the low value for the DoE to achieve potential higher yields using shorter incubation times using higher concentrations. Harvest efficiency was determined both based on DNA measurements and by using a hemocytometer in combination with trypan blue staining, thereby enabling to determine the viability of the resulting cell suspension. Each measurement was performed on triplicate samples obtained from 13 day cultured constructs.

**Table 1:** Experimental conditions used for optimization of Collagenase IV harvest of the expanded cells according to a 3-parameter, 3-level fractional factorial design.

Concentration (U/ml)	Incubation time (hrs)	Flow rate (ml/min)
440	3	0.04
440	5	4
440	7	0.4
660	3	4
660	5	0.4
660	7	0.04
880	3	0.4
880	5	0.04
880	7	4

For the functional characterization of the expanded cell population the optimal harvest condition as determined by the DoE was used. A total of 20 expansion constructs was cultured for 13 days and resulting cell suspensions were pooled for functional characterization of the harvested cells.

### DNA measurement

The DNA content of samples was determined using a highly quantitative and selective DNA assay (Quant-iT dsDNA HS kit, Life Technologies). The constructs were rinsed with PBS and lysed in 350 μl RLT lysis buffer (Qiagen) supplemented with 3.5 μl β-mercaptoethanol after which the lysed samples were vortexed for 60 s and stored at -80°C. Prior to analysis, the samples were thawed at room temperature and spun down for 1 min at 13,000 rpm. 10 μl of the sample was diluted in 90 μl milliQ water after which the DNA content was quantified with a Qubit Fluorometer (Life Technologies) [40]. For measurement of DNA content of the cell suspensions resulting from the harvest procedure the cells were rinsed with PBS and re-suspended in 350 μl RLT lysis buffer supplemented with 3.5 μl β-mercaptoethanol. Further processing for the DNA measurements was identical as described for the constructs.

### Proliferation assay

In order to determine the proliferative capacity of cells expanded in the 3D perfusion bioreactor cells were seeded at a density of 10,000 cells/well in a standard 24-well cell culture plate (Nunc) and cultured for 6 days in CM (n = 4). Proliferation was monitored daily using the Presto Blue metabolic assay by replacing the growth medium with 0.5ml of a 9% Presto Blue solution and incubating for 2 hours. The resulting fluorescent signal was subsequently measured as described before. 2D expanded cells were used as control.

### Osteogenic differentiation assay

Both 2D and 3D expanded cells were seeded at 4,500 cells/cm^2^ in quadruplicate wells and were allowed to proliferate for 2 days prior to adding osteogenic inductive medium (CM supplemented with 100 nM Dexamethasone, 50 μg/ml ascorbic acid and 10 mM β-glycerolphosphate (All from Sigma)) which was subsequently refreshed every two days for 21 days [41]. Cultures were subsequently rinsed with PBS, fixed with 4% formaldehyde and rinsed with distilled water prior to staining with alizarin red solution (pH 4.2) for 60 min. Non-specific staining was removed by extensive rinsing with distilled water after which the calcium deposits were quantified by dissolving the bound dye with 10% cetylpyridinium chloride (in distilled water) for 60 min and measuring the absorbance of the resulting solution at 570 nm.

### Chondrogenic differentiation assay

The chondrogenic differentiation capacity of the 3D and 2D expanded hPDCs was determined based on a micromass assay as described earlier [41]. Briefly, quadruplicate 10 μl micro-masses containing 200,000 cells each were made in 24-well plates for both 2D and 3D expanded cells and incubated overnight in CM. Subsequently the medium was replaced by chondrogenic inductive medium based on DMEM-F12 (Life Technologies) supplemented with 2% FBS, 1% antibiotic–antimycotic, 1X insulin, transferrin, selenous acid (ITS+) Premix universal Culture Supplement (Corning), 100 nM Dexamethasone, 10 μM Y27632 (Axonmedchem), 50 μg/ml Ascorbic Acid, 40 μg/ml Proline (Sigma) and 10 ng/ml transforming growth factor beta 1 (TGFβ1) (Peprotech) [41]. Chondrogenic medium was refreshed every 2 days for 7 days after which the micro-masses were rinsed with PBS and fixed with ice cold methanol for 1 hour at 4°C. Subsequent rinsing steps with PBS and MiliQ water were followed by staining for 1 hour with a 0.1% Alcian Blue solution (in 0.1 M HCl). Non-specific dye was thereafter removed and a 6M guanidine hydrochloride solution was added overnight to dissolve the dye bound to the glycosaminoglycans present and quantification was performed by measuring the resulting absorbance at 620nm.

### Adipogenic differentiation assay

2D and 3D expanded cells were seeded at a density of 10,000 cells/cm^2^ in 24-well plates in quadruplicate and incubated overnight in CM. Subsequently medium was replaced by adipogenic inductive medium based on αMEM (Life technologies) supplemented with 10% FBS, 1% antibiotic–antimycotic, 1μ M Dexamethasone, 10 μg/ml human insulin, 100 μM indomethacin and 25 μM 3-Isobutyl-1-methylcanthine (all from Sigma) [41]. Medium was refreshed every 2 days for a total duration of 14 days after which the cultures were rinsed with PBS and fixed with a 10% formaldehyde solution for 30 min. Plates were subsequently rinsed twice with PBS and dried at room temperature prior to adding the Oil red O staining solution (0.2% Oil Red O in a 60% Isopropanol solution). Samples were stained for 1 hour after which the staining solution was removed, rinsed with PBS to remove non-specific staining and destained for quantification at 492 nm with pure isopropanol.

### In vivo ectopic implantation

*In vivo* bone forming capacity was evaluated using an ectopic implantation model [2]. NuOss, a porous bone mineral matrix material (ACE surgical Sypply CO), and Bio-Oss, a bone substitute for regenerative dentistry (Geistlich), were used as a scaffold material. Cylindrical scaffolds with a diameter and height of 3 mm were punched out of the raw material and drop seeded with 1,000,000 2D or 3D expanded cells in a volume of 25 μl (n = 3 or 4 respectively for the Bio-Oss and NuOss scaffolds). Scaffolds were incubated overnight at 37°C in 3 ml of CM to allow cell attachment after which they were implanted ectopically in the back at the cervical region and the lower back of female NMRI-nu/nu mice as described previously [2]. Empty scaffolds were implanted as negative control. The total of 21 scaffolds were randomly distributed between the 6 experimental animals and grouped per scaffold type. The implants were collected after 8 weeks of implantation and fixed in 4% paraformaldehyde. The animals were sacrificed using cervical dislocation. The volume of mineralized tissue in the explants was quantified using nanoCT as described further on, after which they were decalcified in EDTA/PBS (pH 7.5) for 14 days, embedded in paraffin and processed for haematoxylin and eosin (H&E) as well as Masson’s Trichrome staining. All procedures on animal experiments were approved by the local ethical committee for Animal Research, KU Leuven (P171-2011). The animals were housed according to the guidelines of the Animalium Leuven (KU Leuven). All sections of this report adhere to the ARRIVE Guidelines for reporting animal research [42]. A completed ARRIVE guidelines checklist is included in S1 Checklist.

### nanoCT based quantification of mineralized tissue volume

For visualization and quantification of the mineralized tissue volume the same system was used as for the neo-tissue visualization in the perfusion bioreactor expanded constructs but the tungsten target was operated at a voltage of 60 kV and a current of 210 μA. A 1 mm thick aluminum filter was used to reduce beam hardening and the isentropic voxel size was 3μm.

The resulting transaxial images were analyzed by CTAn (Skyscan NV). Images were segmented into three distinct phases (background, mineralized tissue and scaffold grains) using a 2-Level, 3D multilevel Otsu algorithm generating grayscale images with distinct grayscale values for each fraction. A global threshold was applied to select the scaffold fraction upon which a closing of 1 voxel was applied in combination with a despeckling operation of 200 voxels (black and white) to remove image noise prior to analysis of the scaffold volume. In order to prevent edge effects and the correlated partial volume effect from influencing quantification of the bone volume the selected scaffold fraction was subsequently dilatated with 1 voxel and subsequently subtracted from the original grayscale images. The grayscales corresponding with the newly formed bone were selected and image noise was removed as performed for the scaffold fraction prior to volumetric analysis.

### Statistical analysis

Student t-test was performed to analyse significant differences between individual conditions for data not analysed in the DoE using Statistica 7 (Statsoft). A p-value < 0.05 was considered significant.

## Results

### Perfusion bioreactor facilitated cell expansion

**Fig 1:**
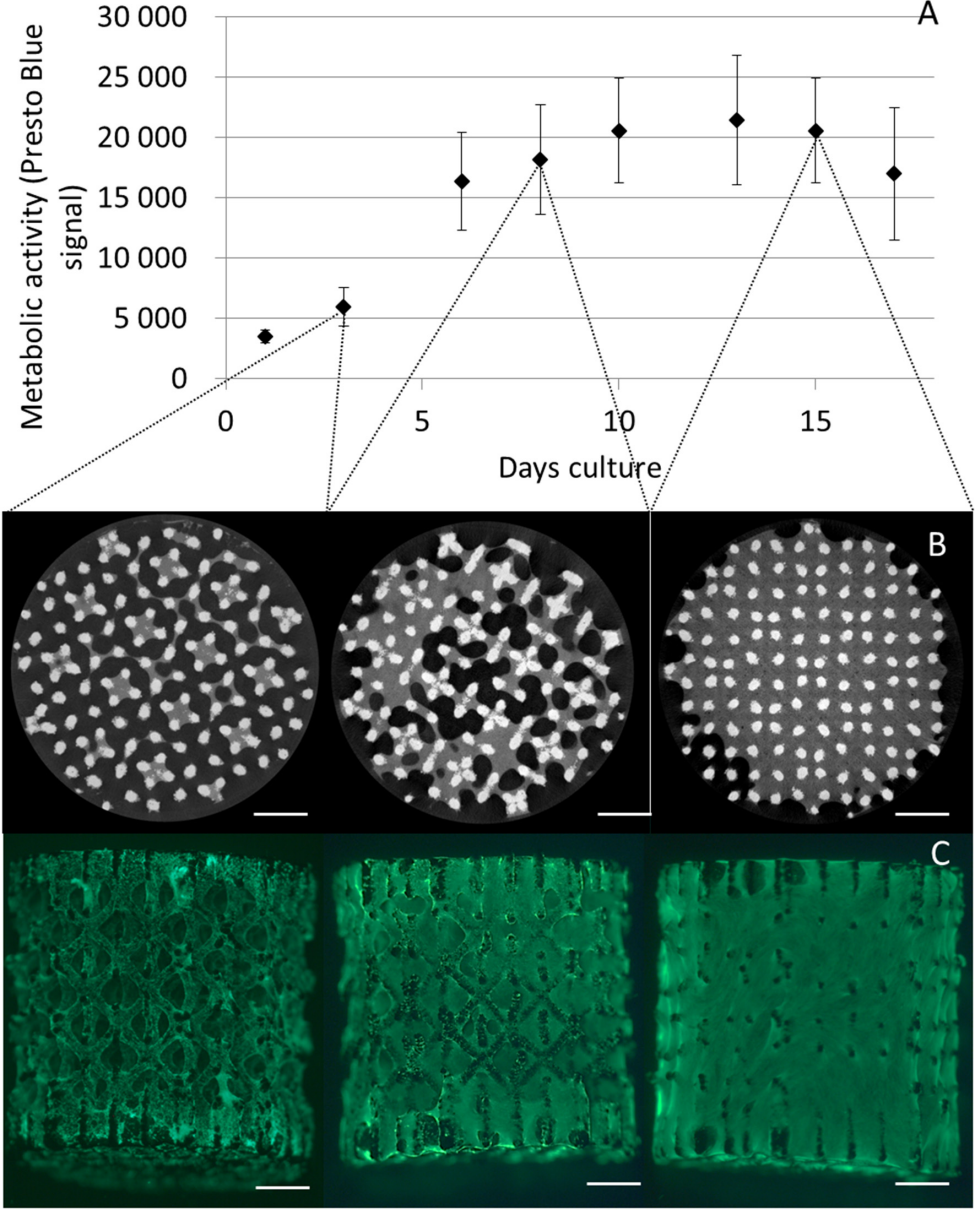
Proliferation and neo-tissue development during 3D perfusion bioreactor cell expansion. (A) Presto Blue based metabolic activity of perfusion bioreactor expanded constructs (n = 4). (B) Contrast Enhanced nano-Computed X-ray Tomography images of constructs at day 3, 8 and 15. Scale bar is 1 mm (C) Calcein AM staining of perfusion bioreactor expanded constructs at day 3, 8 and 15. Scale bar is 1mm.

The Presto Blue metabolic assay was used to monitor hPDC population proliferation during bioreactor expansion in correspondence with earlier published work [32]. A strong increase in metabolic activity was observed during the first 10 days of culture which thereafter levelled off at day 13 to subsequently decreased towards day 17 as shown in Fig 1. Both Calcein AM staining and CE-nanoCT showed that 3D cell growth and ECM deposition gradually filled the voids on the 3D culture substrate, resulting in filled pores towards day 15. Based on the Presto Blue, a 10 fold expansion of the initial cell population was obtained at day 13 with an average cell density of 9,200 cells/mm^3^ or 1,200,000 cells/construct.

### Reagent screening for cell harvest

**Fig 2:**
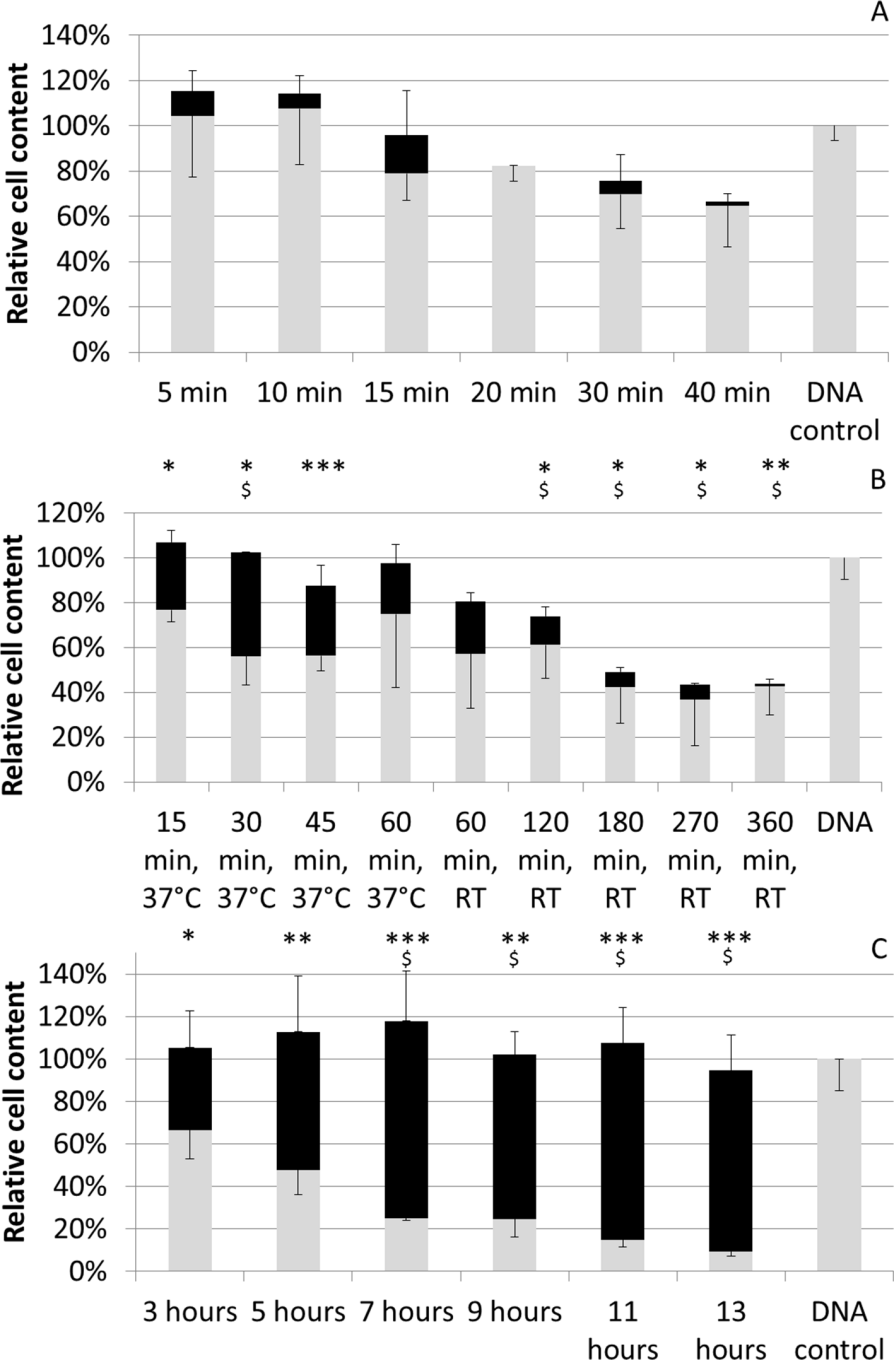
Harvest efficiency using different reagents and incubation times. Harvest efficiency as determined by DNA measurements and shown relative in comparison to non-treated constructs (DNA control). Grey bars depict fraction of the cells remaining on the scaffold, black bars the recovered cell fraction. (A) 0.05% Trypsin—EDTA treatment (B) Accutase treatment at 37°C or room temperature (RT) (C) 440 U/ml Collagenase IV treatment. All procedures were performed at a flow rate of 1ml/min. Significant differences in DNA content of the constructs after treatment in comparison to the DNA control are depicted with *(0.05>p>0.01), ** (0.01>p>0.001) and ***(0.001>p). Significant differences in DNA content of the recovered cell fraction in comparison to what was obtained at the first time point for each treatment are depicted with $. n = 4 for all measurements.

DNA measurements on the scaffolds after harvest showed a significant decrease of cells attached to the 3D culture substrate in function of time for all 3 harvest reagents (Fig 2). Despite the significant, time dependent, decrease in cells remaining on the scaffolds, DNA measurement on the harvested cell suspension did not showed a significant recovery of cells using trypsin (Fig 2A). Accutase enabled the recovery of approximately 23% of cells after 15 minutes of incubation but no time-dependent increase in yield was observed at 37°C. Subsequent testing for longer incubation times at room temperature resulted in significant cell loss (Fig 2B). Using collagenase IV a significant increase in cell recovery up to 76% was observed up to 7 hours of incubation. Prolonged incubation did not result in further significant increases of cell yield and no cell loss was either observed (Fig 2C). Therefore collagenase IV was chosen as reagent for harvest optimization.

### Dynamic harvest optimization

**Fig 3:**
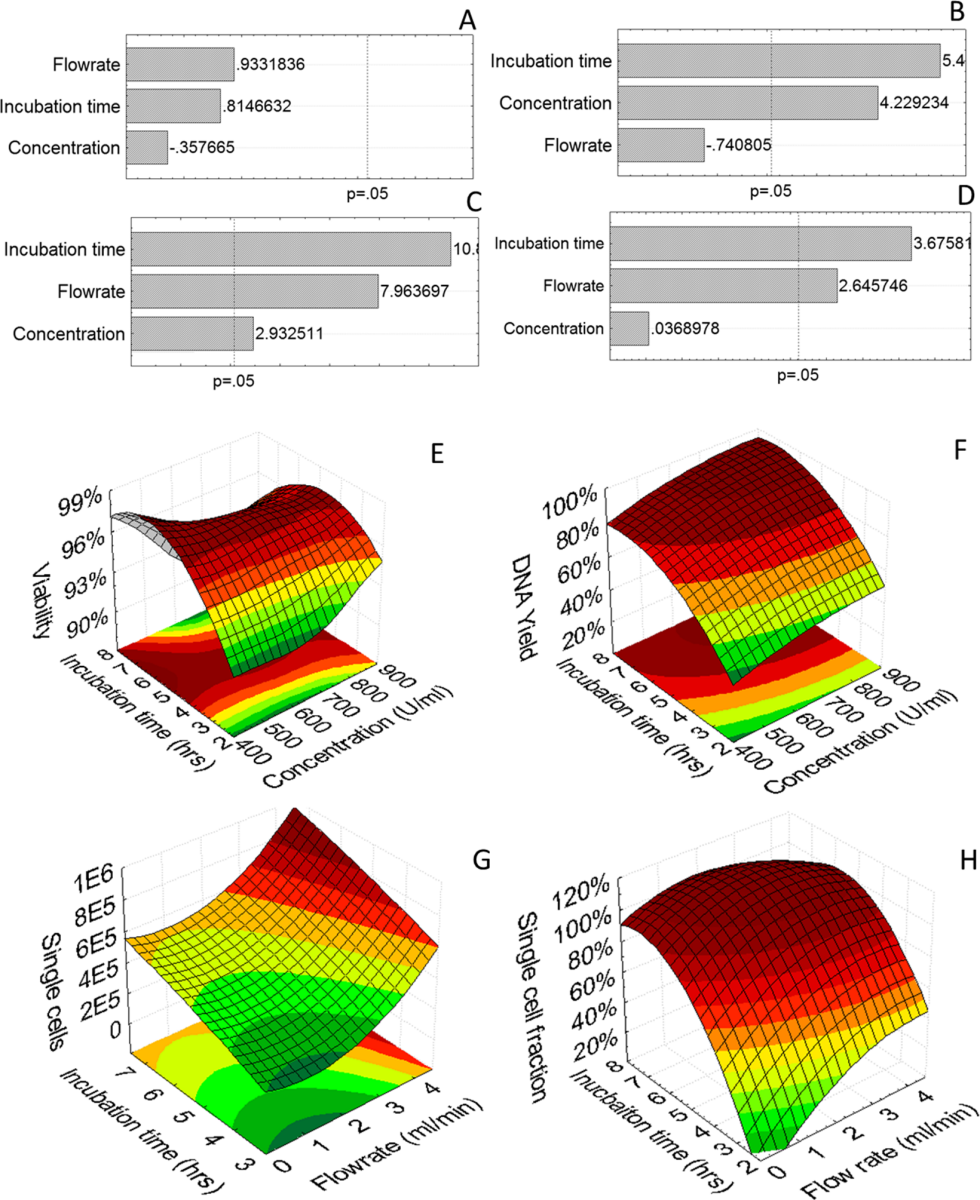
DoE analysis of harvest optimization study using collagenase IV. (A-D) Pareto chart of standardized effects and (E-H) 3D surface plots for respectively (left to right) Cell viability, Harvest yield as determined with DNA measurements, Single cell yield determined by hemocytometer, Single cell content of the recovered cell suspension.

A 3-level, 3-parameter fractional factorial design (Table 1) was employed to study the (i) influence of incubation time, (ii) flow rate and (iii) concentration of collagenase IV on 3D dynamic harvest efficiency. The incubation time did not influence the viability of the harvested cells (Fig 3A and 3E). Additionally, neither collagenase concentration, nor shear stress due to fluid flow were shown to have an influence on the viability of the harvested cell population which was on average 96 ± 2%. Both incubation time and collagenase concentration significantly increased cell recovery as determined by DNA measurements (Fig 3B and 3F). Flow rate was not shown to influence the overall harvest efficiency within the examined range. Incubation time, flow rate and concentration (ranked in order of importance) did however have a significantly positive effect on the single cell yield as determined by manual cell counting (using a hemocytometer) (Fig 3C and 3G). As for certain conditions the presence of cell aggregates was observed in the recovered cell fraction the single cell fraction was also quantified and shown to be positively influenced by an increase in incubation time and flow rate (Fig 3D and 3H).

### In vitro functional assessment of the harvested hPDC population

**Fig 4:**
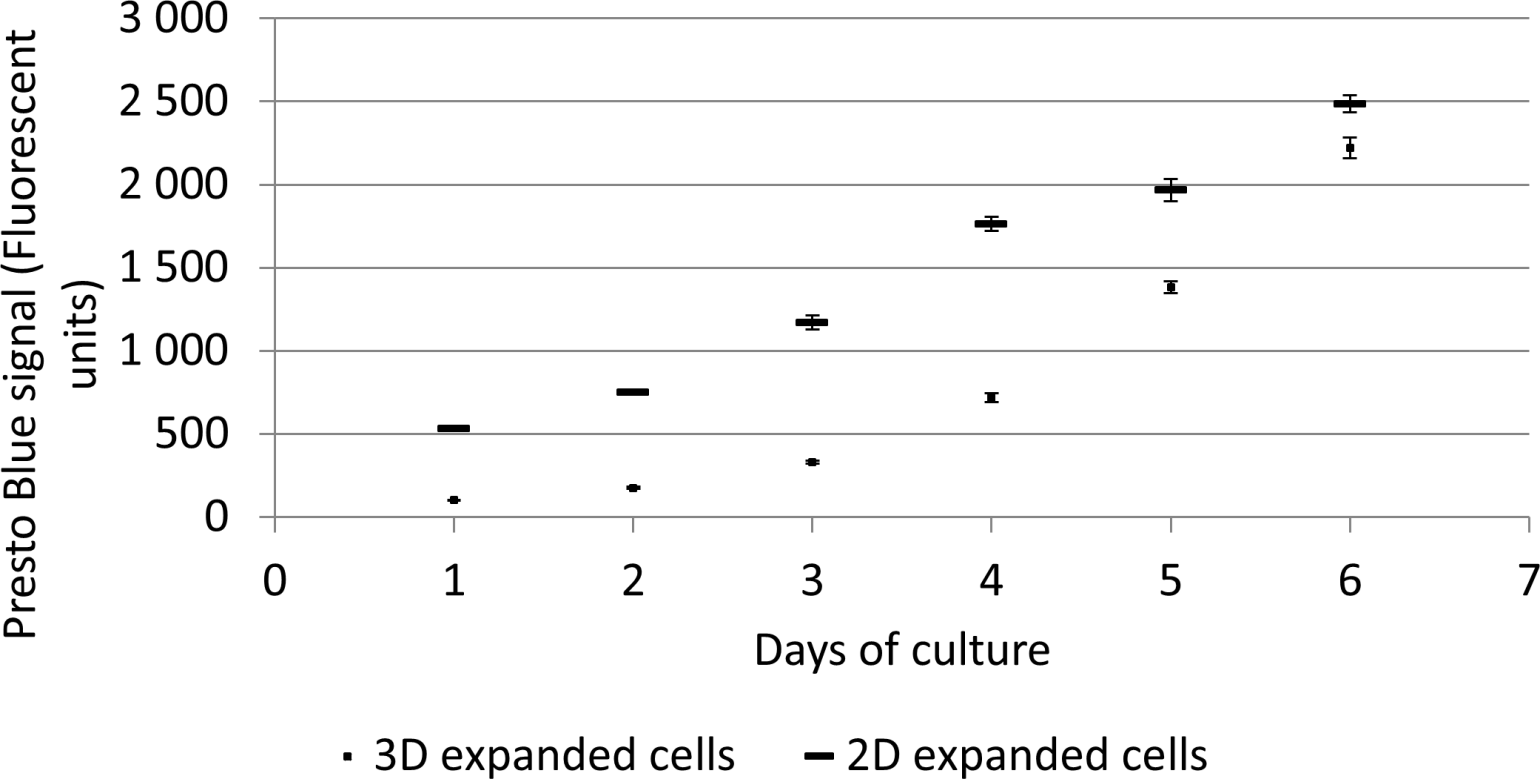
Post-harvest cell proliferation assay. Metabolic activity during 2D proliferation assay of 2D and 3D expanded cells as measured with the Presto Blue. 3D expanded cells were recovered using a 880U/ml collagenase IV solution for 7 hours at a flow rate of 4ml/min. n = 4.

Harvested cells were seeded in well plates to investigate their proliferative potential. The Presto Blue assay was used to monitor cell growth over time showing a significantly lower metabolic activity in the proliferation assay for the 3D expanded cells (Fig 4). The profile of the growth curves was however similar for the duration of the proliferation assay.

**Fig 5:**
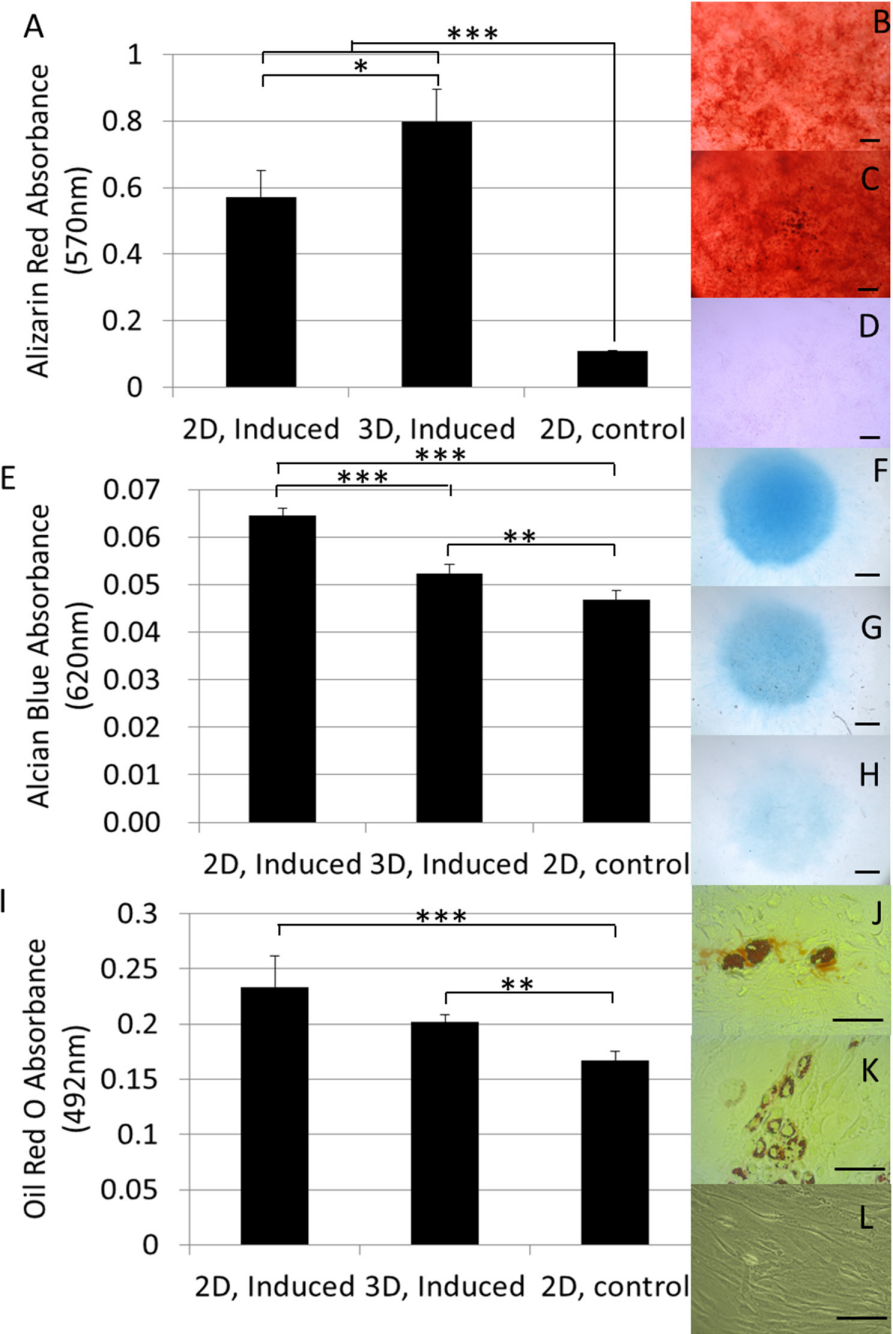
*In vitro* functional assessment harvested cells. (A) Quantification of the calcium depositions in the osteogenic differentiation assay using Alizarin red absorbance at 570nm. (B-D) Alizarin red staining of osteogenic induced hPDCs subsequent to (B) 2D expansion or (C) 3D expansion and (D) non-induced control from 2D expanded cells. (E) Quantification of the glycosaminoglycan deposition in the micromass based chondrogenic differentiation assay using Alcian blue absorbance at 620nm. (F-H) Alcian Blue staining of chondrogenic induced hPDC micromass systems subsequent to (F) 2D expansion or (G) 3D expansion and (H) non induced control from 2D expanded cells. (I) Quantification of the lipid globules formed in the adipogenic differentiation assay using Oil Red O absorbance at 492nm. (J-L) Oil Red-O staining of adipogenic induced hPDCs subsequent to (J) 2D expansion or (K) 3D expansion and (L) non induced control from 2D expanded cells. 3D expanded cells were recovered using a 880U/ml collagenase IV solution for 7 hours at a flow rate of 4ml/min. n = 4 for all samples. Scale bar is 100μm.

Post-harvest tri-lineage differentiation assays showed that the dynamically 3D expanded and harvested cells maintained their *in vitro* differentiation capacity as shown in Fig 5. For the osteogenic differentiation assay a significantly higher amount of mineral deposits was observed in comparison to the 2D expanded cells as well as to the negative control as shown after quantification of the alizarin red staining (Fig 5A). Gycosaminoglycan accumulation induced by chondrogenic differentiation in the micromass system was lower than what was observed for the 2D expanded cells though still significantly higher than for the negative control (Fig 5E). Both 2D and 3D expanded cells also showed a significant capacity for adipogenic differentiation as shown by the lipid globule deposition visualized by Oil Red-O staining and no significant differences were observed between both culture conditions (Fig 5I).

### In vivo functional assessment

**Fig 6:**
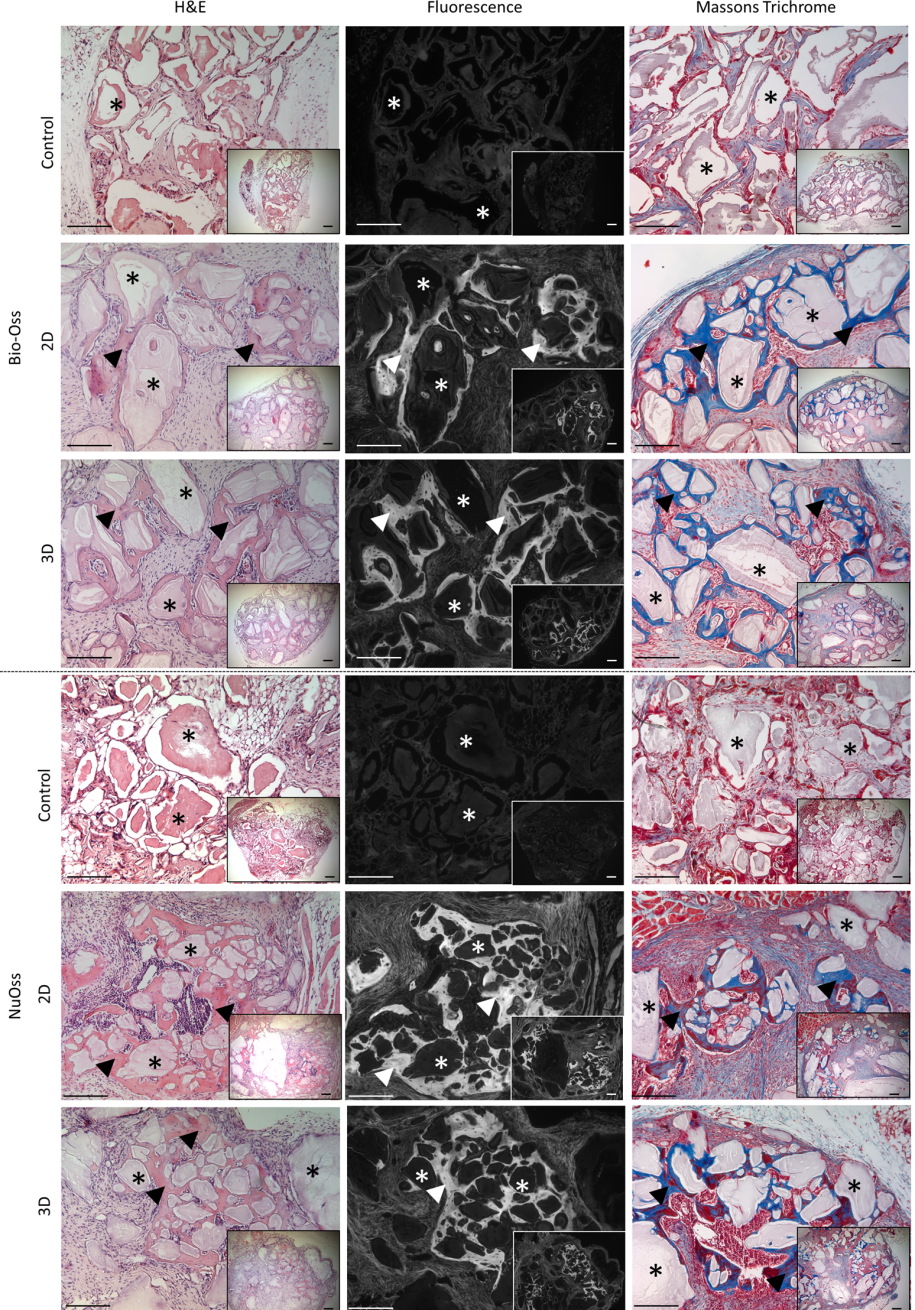
Histological analysis of explants obtained after the *in vivo* functional assessment of harvested cells. Histological sections (H&E, H&E autofluorescence and Masson’s Trichrome) from Bio-Oss and NuOss constructs seeded with 2D and perfusion bioreactor expanded hPDCs respectively as well as the non-cell seeded controls after 8 weeks of *in vivo*. Asterisks indicate remaining Calcium Phosphate grains, arrow heads indicate newly formed bone with embedded osteocytes. Scale bar is 100 μm.

**Fig 7:**
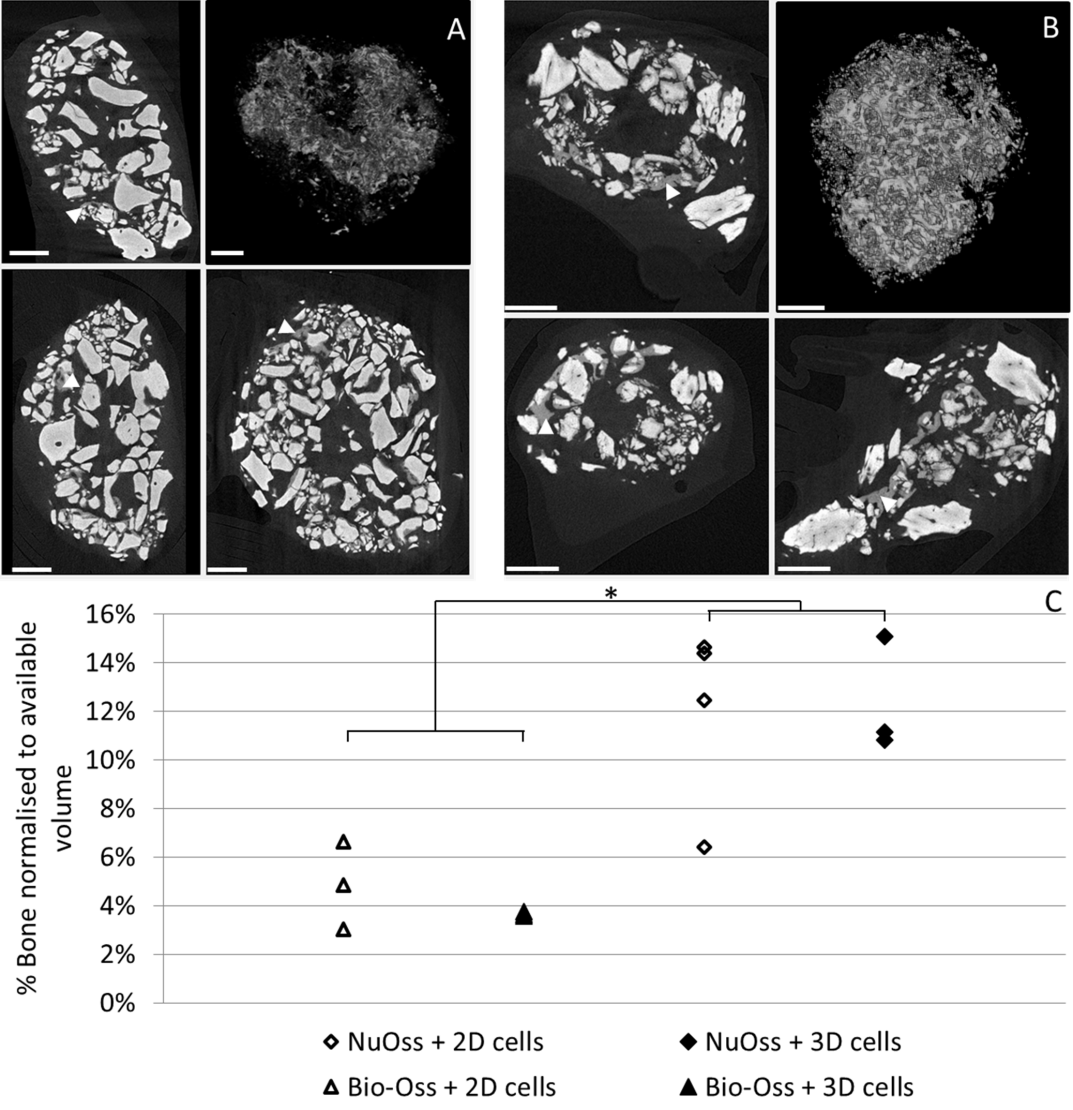
Bone volume analysis using μCT. (A and B) sagittal, transaxial and coronal 2D μCT based section of respectively Bio-Oss (A) and NuOss (B) explant showing CaP grains of the scaffold material (light grey) and newly formed bone (dark grey, indicated with white arrow) combined with 3D visualization of the processed images showing the newly formed bone (right top). Scale bar is 500μm. (C) μCT based quantification of bone formed in the explants after the respective implantation periods normalized to the available volume in the scaffold. 3D expanded cells were recovered using a 880 U/ml collagenase IV solution for 7 hours at a flow rate of 4 ml/min. Scale bar is 100 μm. n = 3 for Bio-Oss constructs, n = 4 for NuOss constructs. * p<0,05.

The *in vivo* bone forming functionality of the expanded hPDC population was evaluated in an established ectopic bone formation model using calcium phosphate–collagen based scaffolds [2]. Both in Bio-Oss and NuOss significant bone formation was observed as determined with H&E and Masson’s Trichrome staining (Fig 6). Quantification of the bone volume was performed based on nanoCT analysis (Fig 7A and 7B) and showed no significant differences between 2D and 3D expanded cells for both scaffold types. Respectively 4.25% and 12.1% of the available volume were filled with bone in the Bio-Oss and NuOss scaffolds (Fig 7C). No significant bone formation could be observed in the non-cell seeded controls for both materials as can be observed in the histological sections (Fig 6). CT-based quantification of the mineralized tissue could therefore also not be performed on these samples.

## Discussion

Despite the need for the development of methods for cell recovery from 3D cell culture substrates this still remains a largely unexplored field [17, 18]. Although recently the use of microcarrier based expansion systems has started to receive more attention, differences between these and other culture setups hamper the translation of existing harvest techniques across setups [18]. We therefore developed and optimized a dynamic methodology that enabled the recovery of cells from a 3D culture substrate after which the functional characteristics of the expanded cell population was assessed and compared to established 2D cultured cells.

Using metabolic activity measurements, which are directly correlated with the total cell number [32], the time-point for cell recovery was set at 13 days to ensure maximum cell expansion and prevent over-confluence (Fig 1). Both the Calcein AM staining and the CE-nanoCT showed that the 3D culture environment was gradually being filled by cells and ECM resulting in 3D confluency by day 15 where no further hPDC expansion was expected [11, 12]. The decrease of metabolic activity measurements at subsequent time points further indicated that cell growth ceased at this time point.

In order to choose the most suitable harvest reagent for cell recovery the use of Trypsin/EDTA, Accutase and collagenase IV was evaluated (Fig 2). Trypsin and Accutase are commonly used reagents for cell recovery from 2D cell culture substrates on which cells grow predominantly in a monolayer such as standard 2D cell expansion systems or microcarriers [24, 29, 43–45]. The loss of cells observed using trypsin and Accutase (respectively up to 35% and 57%) could be explained by the mechanism by which they act. Both agents are known to specifically cleave peptide chains at the carboxyl site of lysine or arginine. They will therefore cleave extracellular proteins as well as trans-membrane associated structures leading to the time dependent increase in cell loss observed for both solutions. Most setups (always 2D) therefore use a maximal incubation time of 10 to 15 minutes [19–21]. Additionally, trypsin it is known to be unable to cleave the helical structures of the collagen fibers, hence limiting its capacity to degrade the *in vitro* deposited collagen. As this is a major constituent of the ECM its application for cell recovery from a 3D cell culture environment within which abundant ECM is present is therefore suboptimal. Prolonging the incubation with trypsin in our system did consequently not result in an increased release of the cells from the deposited ECM but, as shown in Fig 2, only resulted in progressive damage to the expanded cell population. The use of a pure collagenolytic solution was expected to prevent this proteolytic related cell degradation as these enzymes specifically target the peptide bounds in collagen. As this is a major constituent of the ECM but is not present in the cell membranes it was successfully used previously for the recovery of primary cells from various tissues [26, 46, 47] and application in our system resulted in a successful recovery with an overall cell recovery efficiency of up to 92% (Fig 2C).

Expanding further on the successful application of the collagenase IV solution for the recovery of single cells from the 3D culture substrate a DoE approach was employed to optimize its use. Prolonged exposure to collagenase (between 10 and 24 hrs) was previously shown to affect cell viability for the digest of primary tissues [26, 48], and as the strongest increase in yield was observed during the first 7 hours in the screening experiments (Fig 2C), this was chosen as the maximal incubation time for the DoE study. None of the studied parameters showed a linear influence on the viability of the recovered cell population within the examined operating window which was on average 96% (Fig 3A and 3E). Concentration and incubation time were shown to have a significant effect on both overall harvest efficiency and single cell yield while flow rate only had a significant positive effect on the single cell yield (Fig 3B, 3C, 3F and 3G). The single cell fraction was positively influenced by a high incubation time and flow rate resulting in a continued dissociation of the aggregates into a single cell suspension as was shown earlier for different stem cell aggregate systems (Fig 3D and 3H)[49]. No adverse effects related to shear stress on cell viability were observed in accordance with previous studies for comparable flow regimes as the maximal shear stress experienced by the cells in the tubing was approximately 2 mPa [50, 51]. This was evidenced by unaffected single cell viability while the cumulative DNA content of the cells remaining on the 3D culture substrate and in the harvested suspension was equal to that of the post expansion, pre-harvest culture environment (as also shown in Fig 2C).

Despite the fact that no adverse effects of the applied procedure on the cell viability were detected the metabolic activity of the hPDCs was significantly decreased after replating in comparison to standard 2D expanded cells (Fig 4). Adverse effects of prolonged use of a collagenase solution between 10 and 24 hours were reported for the digest of primary tissues which could result in a decrease of the metabolic activity of the harvested cell population [26, 48]. Recent work also showed cells to possess a “mechanical memory” which can influence their behavior depending on the previous physical environments used for culture [52]. The switch from the complex, ECM containing 3D culture environment in the perfusion bioreactor system to the standard tissue culture plastic might therefore also influence the metabolic activity of the recovered hPDC cell population and might result in an initial lower metabolic activity.

Maintenance of the *in vitro* differentiation potential of a progenitor cell population expanded in bioreactor systems has been reported for several MSC cell sources and was also observed in our system (Fig 5) [10, 24, 53]. The observed increase in calcium depositions and decrease in GAG accumulation suggest an osteogenic priming of the expanded cell population (Fig 5). In previously published work we already hypothesized that the shear stress exerted on the cells during expansion might result in osteogenic priming of the expanded cell population based on the up regulation of certain gene markers associated with osteogenic differentiation which is further substantiated by this data [11]. Although the 3D perfusion bioreactor expansion of primary MSCs was previously shown to result in enhanced *in vitro* osteo-, chondro- and adipogenic differentiation potential as well as an enhanced clonogenicity of freshly isolated primary MSCs [10], we only observed an increase in osteogenic differentiation (Fig 5A). The cells used for this study were however pre-expanded for multiple passages using standard cell culture methodologies, including the use of plasma treated polystyrene. This has been correlated with the progressive loss of the progenitor cell phenotype and commitment to the osteogenic lineage indicating that the differences in post expansion differentiation potential for primary cells might originate from an enhanced maintenance of the progenitor cell phenotype [7–9]. The initial 2D expansion of the hPDC population used for this work could therefore explain the absence of a significant difference in differentiation potential between 2D and 3D expanded cells in this culture setup.

Post expansion cell characterization is currently focused on cell identity as established by *in vitro* assays [10, 24, 53] while their functionality as evidenced by their performance in both *in vitro* and *in vivo* models is largely subsided. Furthermore the lack of such data hinders the development of correlations that will eventually allow to link *in vitro* and *in vivo* cell behavior [31]. To the best of our knowledge no reports exist assessing the *in vivo* potency of a progenitor cell population after expansion and recovery in a 3D bioreactor system despite the need for adequate *in vivo* controls for the validation of these processes [31]. We therefore assessed the *in vivo* bone forming capacity of the 3D expanded and recovered cells in a standard ectopic bone formation assay [2]. Histological staining showed the clear presence of bone spicules in all cell seeded conditions (Fig 6). In addition, the presence of bone marrow lacunae and blood vessels was unimpaired in comparison to their 2D control indicating the unaffected *in vivo* differentiation capacity of the 3D expanded cells. This was further confirmed and quantified by nanoCT analysis where no differences between 2D and 3D expanded cells were apparent (Fig 7C) and observed volumes of newly formed bone were comparable to what was shown earlier [2].

In conclusion, we optimized a closed bioprocess enabling the expansion and recovery of a viable cell population. The produced cell population showed minor differences in its *in vitro* differentiation potential as compared to the 2D standard while it showed an uncompromised bone forming potential upon ectopic implantation. The development of this multi-unit integrated bioprocess shows the potential for integrated bioprocess development and sets the scene for the continued integrated optimization of current manual tissue engineering strategies to a clinical relevant setting.

## Supporting Information

S1 Checklist Completed “ARRIVE Guidelines Checklist” for the animal data reported in this manuscript. (PDF)
